# Field-Evolved Resistance in Corn Earworm to Cry Proteins Expressed by Transgenic Sweet Corn

**DOI:** 10.1371/journal.pone.0169115

**Published:** 2016-12-30

**Authors:** Galen P. Dively, P. Dilip Venugopal, Chad Finkenbinder

**Affiliations:** 1 Department of Entomology, University of Maryland, College Park, Maryland, United States of America; 2 American Association for the Advancement of Science - Science and Technology Policy Fellowship Program, Transportation and Climate Division, Office of Transportation & Air Quality, United States Environmental Protection Agency, District of Columbia, United States of America; 3 Benzon Research Inc., Carlisle, Pennsylvania, United States of America; University of Tennessee, UNITED STATES

## Abstract

**Background:**

Transgenic corn engineered with genes expressing insecticidal toxins from the bacterium *Bacillus thuringiensis* (Berliner) (Bt) are now a major tool in insect pest management. With its widespread use, insect resistance is a major threat to the sustainability of the Bt transgenic technology. For all Bt corn expressing Cry toxins, the high dose requirement for resistance management is not achieved for corn earworm, *Helicoverpa zea* (Boddie), which is more tolerant to the Bt toxins.

**Methodology/Major Findings:**

We present field monitoring data using Cry1Ab (1996–2016) and Cry1A.105+Cry2Ab2 (2010–2016) expressing sweet corn hybrids as in-field screens to measure changes in field efficacy and Cry toxin susceptibility to *H*. *zea*. Larvae successfully damaged an increasing proportion of ears, consumed more kernel area, and reached later developmental stages (4^th^ - 6^th^ instars) in both types of Bt hybrids (Cry1Ab—event Bt11, and Cry1A.105+Cry2Ab2—event MON89034) since their commercial introduction. Yearly patterns of *H*. *zea* population abundance were unrelated to reductions in control efficacy. There was no evidence of field efficacy or tissue toxicity differences among different Cry1Ab hybrids that could contribute to the decline in control efficacy. Supportive data from laboratory bioassays demonstrate significant differences in weight gain and fitness characteristics between the Maryland *H*. *zea* strain and a susceptible strain. In bioassays with Cry1Ab expressing green leaf tissue, Maryland *H*. *zea* strain gained more weight than the susceptible strain at all concentrations tested. Fitness of the Maryland *H*. *zea* strain was significantly lower than that of the susceptible strain as indicated by lower hatch rate, longer time to adult eclosion, lower pupal weight, and reduced survival to adulthood.

**Conclusions/Significance:**

After ruling out possible contributing factors, the rapid change in field efficacy in recent years and decreased susceptibility of *H*. *zea* to Bt sweet corn provide strong evidence of field-evolved resistance in *H*. *zea* populations to multiple Cry toxins. The high adoption rate of Bt field corn and cotton, along with the moderate dose expression of Cry1Ab and related Cry toxins in these crops, and decreasing refuge compliance probably contributed to the evolution of resistance. Our results have important implications for resistance monitoring, refuge requirements and other regulatory policies, cross-resistance issues, and the sustainability of the pyramided Bt technology.

## Introduction

Transgenic crops engineered with genes expressing insecticidal toxins from the bacterium *Bacillus thuringiensis* (Berliner) (Bt) are now a major tool in insect pest management, with increasing global adoption since commercial availability in 1996 [1]. Bt transgenic technology is safe for human health, and may provide multi-fold benefits including reductions in pests and pesticide usage, and increases in yield, profits and food security [2–9]. In the U.S., single-gene and pyramided Bt crops constituted 81% of the total 35.6 million ha of maize (corn), *Zea mays* L., planted in 2015, and 84% of the total 3.6 million ha of planted cotton *Gossypium sp*. L. [10,11]. Bt toxins targeting lepidopteran pests in these crops include Cry1Ab, Cry1Ac, Cry1A.105, Cry1F, Cry2Ab2, Cry2Ae and Vip3A.

With the widespread use of Bt crops, insect resistance is the major threat to the sustainability of the Bt transgenic technology. Thus, an insect resistance management (IRM) plan aimed at delaying the onset of resistance is required by the U. S. Environmental Protection Agency (EPA) for commercial registration of Bt crops in the U.S [12]. As the key component of IRM, high dose-refuge strategies have been widely adopted targeting specific insect pests, which deploy a high toxin expression to kill nearly all heterozygous resistant individuals. When used in combination with non-Bt crops grown in proximity or amidst Bt crops as refuges, this strategy allows survival and mating of susceptible insects with Bt resistant insects, thereby keeping the resistant allele frequency low [13]. For single-gene Bt corn hybrids, primarily targeting European corn borer, *Ostrinia nubilalis* (Hübner), EPA mandates a 20% and 50% structured refuge in the corn belt and cotton growing regions, respectively. Also, the refuge-in-the-bag (RIB) strategy with a 5% refuge requirement (95% Bt) is now approved for hybrids expressing multiple pyramided Bt proteins for lepidopteran control in the corn belt [14]. The RIB strategy primarily aims to manage evolution of resistance in *O*. *nubilalis* (Hübner), but not corn earworm, *Helicoverpa zea* (Boddie). For this reason, there still is a 20% structured refuge requirement for Bt corn with no option for the RIB strategy in the southern U.S. which faces high *H*. *zea* population pressure [15] and additional selection from Bt cotton.

While IRM for Bt corn has proved effective in delaying *O*. *nubilalis* resistance, reports of other insect pests developing resistance to the insecticidal toxins are on the rise (see [16,17] for recent reviews). Since 2002, field-evolved resistance to Bt crops [*sensu* [16,18]; > 50% resistant individuals and reduced efficacy] is reported for five major insect pests across different countries [17]. This includes the fall armyworm, *Spodoptera frugiperda* (J.E. Smith) for Cry1F in Puerto Rico, Brazil and continental U.S. [19–23] and Cry1Ab in Brazil [24]; maize stalk borer, *Busseola fusca* (Fuller) for Cry1Ab in South Africa [25]; pink bollworm, *Pectinophora gossypiella* (Saunders) for Cry1Ac in India [26]; Western corn rootworm, *Diabrotica v*. *virgifera* LeConte for Cry3Bb and mCry3A in U.S. [27–29]; and corn earworm, *H*. *zea* (Boddie) for Cry1Ac in U.S [16, 30]. Reports of field-evolved resistance by *H*. *zea* to Cry1Ac was refuted by Moar *et al*. [31], to which Tabashnik *et al*. [32] responded with further confirmations on the initial report. *H*. *zea* may also be developing resistance to Cry1Ab in field corn [33]. Additionally, cross-resistance can occur among closely related Cry toxins, particularly those selected for pest resistance in single-gene Bt crops, which can likely lead to more rapid pest resistance development in crops expressing multiple pyramided toxins [22,34–37]. Previous reports have shown cross-resistance between the Cry1A proteins (1Ab, 1Ac, Cry1A.105) in *H*. *zea* [38,39]. Santos-Amaya *et al*. [40] found that Cry1F resistant *S*. *frugiperda* selected in Bt corn was also highly resistant to Bt cotton expressing Cry1Ac and Cry1f toxins, and Yang *et al*. [41] reported that a laboratory selected strain of *S*. *frugiperda* to Cry1A.105 and Cry2Ab2 showed cross-resistance to Cry1F. Recently, Hernandez-Rodriguez et al [42] demonstrated that *O*. *nubilalis* and *S*. *frugiperda* shared midgut binding sites for Cry1A.105, Cry1Ab and Cry1Ac, implying cross resistance between these proteins.

Registrants of Bt corn are also required by the EPA to annually monitor potential changes in susceptibility of target insect species to Bt toxins in order to detect the evolution of resistance before field efficacy fails [43]. To monitor susceptibility changes, the performance and mortality of the progeny of field-collected insects to a Bt toxin is tested at diagnostic concentrations, and compared to a known range of baseline susceptibility [44–46]. A significant decrease in susceptibility of a population based on this approach is viewed as genetically mediated and confirmation of field-evolved resistance, as defined by Tabashnik *et al*. [43]. However, when field populations evolve resistance to Bt toxins, decreased susceptibility to Bt crops, resulting in a reduction in field efficacy is usually expected [43]. Moar *et al*. [31] defined field-evolved resistance based on a change in field efficacy, documented as an increased ability of a target pest to feed and complete development. Such a definition of field-evolved resistance incorporates the outcomes of genetically mediated changes in sensitivity of the target pest to Bt toxins, such as the potential for incomplete resistance and fitness costs where pest feeding on Bt crops increases but development to adult is delayed or incomplete [31]. In addition to the laboratory bioassays to determine resistance, comparison of target pest susceptibility and control efficacy in paired fields of Bt and non-Bt crops is a practical method to monitor evolution of resistance in the field [31,47]. Resistance definition by Tabashnik *et al*. [16,18], which we follow in this study, comprises different categories of resistance, including the field efficacy changes as a basis for resistance development.

*H*. *zea* is the key pest of sweet corn and polyphagous in many agricultural crop systems, and it is capable of migrating long distances [15]. For sweet corn production, Attribute (expressing Cry1Ab toxin, event Bt11) and Attribute II (expressing Cry1Ab and Vip3A, event MIR162) hybrids from Syngenta Seeds, and Performance Series (PS) hybrids (expressing the Cry1A.105 and Cry2Ab2 toxins, event MON89034) from Seminis Seeds are commercially available. For these Bt hybrids, which represents <1% of the corn hectares grown in the U.S., there is no refuge requirement. Growers are required to destroy the stalks following harvest. For all Bt corn expressing Cry toxins, the high dose requirement for IRM is achieved for *O*. *nubilalis*, but it is not true for *H*. *zea* which is more tolerant to the Bt toxins [48]. In 2003, Horner *et al*. [49] reported that Bt field corn (Cry1Ab, event MON810) suppressed the establishment and development of *H*. *zea* to late instars by at least 75%. They suggested that this moderate dose effect might increase the risk of evolution of resistance in areas where Cry1Ab expressing corn is widely adopted and *H*. *zea* overwinters successfully. Efficacy of single event Cry1Ab sweet corn for controlling *H*. *zea* was highly variable during 2008–2011, (for example, 8.0%– 73% clean ears in Maryland; [50,51]) with increasing concerns over lack of *H*. *zea* control by Cry1Ab in Bt sweet corn [51].

Here we present findings from 21 years of monitoring changes in Cry1Ab susceptibility to *H*. *zea* and field efficacy in Bt sweet corn as an in-field screen. We hypothesized that a change in control efficacy since its commercial availability in 1996 is evidence of field-evolved resistance of *H*. *zea* to Cry1Ab toxins. We also provide data on recent changes in field efficacy of Cry1A.105+Cry2Ab2 sweet corn, suggesting resistance development in *H*. *zea* to multiple Cry toxins. Further, we investigated patterns of *H*. *zea* population abundance, and differences among Bt hybrids for the efficacy parameters (as surrogate for toxin expression among Bt hybrids) as they might affect control efficacy. Finally, supportive information from laboratory bioassays provide preliminary evidence of susceptibility changes by comparing weight gain, toxicity responses, and fitness characteristics of a *H*. *zea* strain reared from surviving larvae on Bt sweet corn with a susceptible strain.

## Methods

### Ethics statement

No endangered or protected species were involved in the study. Study was conducted at the University of Maryland Research and Education farm facilities for which we had permission to access and collect data.

### Study design

Venette *et al*. [47] proposed the use of sentinel Bt sweet corn as an in-field screen to monitor evolution of target pest resistance and overall efficacy of Bt corn. Sweet corn, *Zea mays* var. *saccharata*, represents an ideal host plant for monitoring early shifts in *H*. *zea* susceptibility and can effectively function as an in-field diagnostic dose for several reasons. First, when first introduced commercially, both Attribute and PS hybrids provided greater than 95% control efficacy against *H*. *zea* in most field trials [50–53]. Second, late season plantings of sweet corn are highly attractive to *H*. *zea* moths during the silking and ear development period, and significantly more infested than populations found in field corn, thus reducing the sample size of ears required to statistically detect changes in *H*. *zea* damage. For late plantings devoid of insecticidal treatments, infestation of most ears of non-Bt hybrids by *H*. *zea* is likely [47]. Unlike field corn, sweet corn is harvested at a premature stage, thus toxin expression is consistently high throughout the crop cycle and generally higher in the silk and kernel tissues than in Bt field corn. Lastly, it is relatively easy to quantify changes in the incidence and severity of ear damage in sweet corn as a measure of control efficacy.

Sentinel plots of Bt sweet corn hybrids paired with non-Bt isogenic hybrids were established each year during 1996 to 2016 to evaluate *H*. *zea* infestations in ears as a direct measure of control efficacy and indication of changes in Bt toxin susceptibility. Cry1Ab hybrids were commercially available in 1996 and evaluated during all years, whereas Cry1A.105+Cry2Ab2 hybrids were developed later and included in studies from 2010 to 2016. Additionally, we evaluated the field performance of pyramided Cry1Ab+Vip3A hybrids in plots planted each year since 2008, alongside other Bt sweet corn types at the same locations. During each year, one or two late plantings were established at five University of Maryland Research and Education farm facilities—Salisbury and Queenstown on the Eastern Shore of Maryland, and at Upper Marlboro (38.86° N, 76.78° W), Beltsville (39.01° N, 76.83° W) and Clarksville (39.25° N, 76.93° W) in central Maryland. Plots were seeded during June at each farm location to encourage high infestations of *H*. *zea* that typically occur in late August during silking and ear development [54,55]. One replicate pair of Bt and non-Bt plots were established at most locations; however, replicated plots of each hybrid were included during some years and locations. Plots ranged in size from 6 to 12 rows 15–30 m long and were maintained according to commercial production recommendations, except no insecticides were applied.

### Control efficacy assessments

All plots were sampled to assess ear damage during mid-August through mid-September when ears reached fresh market maturity (usually 18–21 days after the onset of silking). We examined 50–100 primary ears from the center rows of each plot, depending on plot size and level of infestation. Ears were removed and either brought back to the laboratory for processing or data were recorded directly from husked ears *in situ*. The following variables were recorded as measures of control efficacy: percentage of ears damaged by *H*. *zea*, mean kernel area consumed, and mean instar stage (henceforth damage, consumption, and instar, respectively). Each ear was carefully examined for kernel injury and recorded by pest species. The total kernel area consumed (cm^2^) was visually estimated as a measure of the extent of ear damage. Technicians were trained to estimate injured kernel area to ensure consistent and accurate data. A convenient reference used for estimation was the 0.5 cm^2^ cross-section of a standard pencil eraser. Damage greater than 0.5 cm^2^ was recorded to the nearest 0.5 cm^2^, but for very minor injury on a few kernels (<0.5 cm^2^; common on Bt ears), damage was recorded as 0.2 cm^2^. For calculating mean kernel consumption, we used only the data from damaged ears. The number of live and dead *H*. *zea* larvae found in each ear was recorded by instar. For ears with kernel damage but no larvae present, we assumed a late instar *H*. *zea* completed development if kernel area consumption was greater than 8 cm^2^ with heavy deposits of frass, discarded head capsules, and presence of an exit hole. In addition to the mean instar stage, we calculated the proportion of late instar (4th– 6th instars) found in Bt ears as another measure of larvae development. Fall armyworm, *S*. *frugiperda* (J.E. Smith), is not a major ear-invading pest in Maryland. However, when there was evidence of multi-species infestations without larvae present, we carefully examined for shed head capsules, differences in frass deposits, and characteristic feeding patterns to distinguish between *S*. *frugiperda* and *H*. *zea* damage. *O*. *nubilalis* damage was easily distinguished from that of *H*. *zea* by the characteristic frass and the tunneling injury going into the cob at the tip, side, or base of the ear.

For plantings with replicate plots of Bt and non-Bt hybrids, we used averages over the replicates to avoid pseudo-replication and conducted statistical analyses for each control efficacy variables (damage, instar, consumption and proportion of late instars). We analysed the comparative trends in each variable between Bt and non-Bt hybrids of each Bt type over the study period, except for the Cry1Ab+Vip3A sweet corn data which contained almost all zeros for each variable. Each analysis performed individual linear mixed models (LMM) based on restricted maximum likelihood (REML) assuming a normal error distribution except for proportion of large instar for Cry1A.105+Cry2Ab2 which was analysed through Gaussian GLMM (identity function). Statistical significance of the fixed effects in the LMMs was tested through Wald F tests with Kenward-Roger approximation, and for GLMM through Wald Χ^2^ test [56].

In each of these LMMs, damage, instar, consumption, and proportion of later instars were the response variables, interaction effect of year and treatment (Bt vs non-Bt) was the fixed effect, and the study site was included as a random effect to account for repeated measurement [56]. Where applicable, we square root transformed the data to conform to linear model assumptions. We tested for the interaction effect of year and treatment because it denotes significant slope differences between Bt and non-Bt hybrids.

### Bioassays for resistance characterization in *Helicoverpa zea* field-collected populations

#### Insect collections

Previously we had unsuccessfully attempted to establish a *H*. *zea* colony from larvae surviving Bt sweet corn during 2008–2012 to determine the extent of field-evolved resistance through laboratory bioassays. Procedural details and results of these attempts are available in S1 Appendix. In 2015 we collected surviving *H*. *zea* from two 0.2 h fields of Attribute sweet corn (hybrid ‘BC0805’, expressing Cry1Ab) at two University of Maryland Research and Education facilities. One field was planted on 25 June at Salisbury (38.37° N, 75.66° W), while the second was planted on 20 June at Queenstown (38.80° N, 76.17° W). Fields were planted late so that the attractive silking stages coincided with peak moth activity during late August. Each field was maintained according to commercial production recommendations, except no insecticides were applied. At about 18 days after first silking (late August), ears were husked open *in situ* at both locations to expose surviving *H*. *zea* larvae. We removed 1,200 5^th^ and 6^th^ instars from ears, transferred them to 1 oz plastic cups containing 1.5 ml of *H*. *zea* meridic diet, and brought back to the laboratory where they were reared individually at 25°C until pupation. Pupae were removed and surface sterilized in a 5% Clorox (8.25% sodium hypochlorite ai) solution for 3 min and rinsed in water. In September 2015, 600 pupae were shipped to Benzon Research Inc. (Carlisle, PA), where a breeding colony (henceforth UMD 2015 strain) was established and maintained using standard rearing methods [57] through two generations before bioassays were conducted. The Benzon laboratory also maintains a colony of susceptible *H*. *zea* strain that was used as a standard reference.

#### Tissue-incorporated bioassays

Green leaf tissue was collected from Attribute sweet corn (hybrid ‘BC0805’) and its non-expressing isogenic hybrid ‘Providence’ at full tassel at the Beltsville study site. Two leaves above the ear were removed from a random sample of 20 plants of each type, cut into smaller sections, and dried as a composite sample in a freeze dryer. The lyophilized Bt and non-Bt tissue was then ground to a fine powder in a commercial grinder (IKA Works, Inc, Wilmington, DE) and kept at -80°C until used in bioassays. Eggs of the UMD 2015 and susceptible strains were obtained from Benzon Research and incubated in a growth chamber until hatch. Because eggs of the UMD 2015 strain exhibited delayed development, temperature regimes were manipulated to schedule larvae of the same size of each strain. The neonates were reared on a *H*. *zea* meridic diet (Southland Products, Lake Village, AR) until the early 2nd instar for testing.

Three bioassays, each with two replicates of eight concentrations of Bt leaf tissue, were conducted on different days (4 Feb, 23 Feb and 7 April), using larvae from the 3rd, 4th and 5th generations of the UMD 2015 strain, respectively. At each bioassay, 1200 ml of meridic diet (adjusted with more water to offset for the added leaf tissue) was prepared and cooled to 55°C. in a water bath. Pre-weighed quantities of the Bt and non-Bt powdered tissue were prepared in the following proportions: 1) 300 mg non-Bt, 2) 10 mg Bt plus 290 mg non-Bt, 3) 20 mg Bt plus 280 mg non-Bt, 4) 40 mg Bt plus 260 mg non-Bt, 5) 80 mg Bt plus 220 mg non-Bt, 6) 160 mg Bt plus 140 mg non-Bt, 7) 300 mg Bt, and 8) 600 mg Bt. With the exception of the last quantity, the amount of total leaf powder added to the diet was the same for each concentration to standardize the composition of the diet.

For each diet concentration, 25 ml of molten diet was drawn into a 60 cc syringe with a 4 ml dia opening at the tip. The cap was then placed over the tip and the plunger was pulled back and removed from the syringe. Starting with the non-Bt concentration, a pre-weighed quantity of the leaf powder was added to the diet through the open end of the syringe. The plunger was then re-inserted, and the mixture homogenized by shaking the syringe for 30 seconds, followed by firmly holding the syringe on a rubber platform of a vortex mixer for another 30 seconds. With the tip cap removed, the leaf powder-diet mixture of each concentration was dispensed to one 32-well section of a 128-well bioassay tray (C-D International). Approximately 1.5 ml of diet was added to each well. The dilutions by adding leaf tissue to 25 ml of diet resulted in relatively low exposure doses; for example, the diet incorporating 600 mg of powder contained approximately 2–3% Bt leaf tissue.

After the diet mixture cooled and solidified, one early 2nd instar (18–24 hours old) was placed in each well using a camel-hair brush. Each 16-well section of the bioassay tray was sealed with a perforated adhesive lid. The trays were held in a growth chamber at 25°C, L:D 14:10, and 40–60% RH. Each tray contained one replicate of the eight concentrations and was infested with larvae from either the UMD 2015 or susceptible *H*. *zea* strain. After seven days, live larvae within each row of four wells were pooled and weighed together. The average weight gain per larva was calculated by dividing the pooled weight by the number of larvae in the row group.

Leaf tissue-incorporated bioassays data was analysed through a quasi-Poisson GLM with average weight after a week of feeding as the response variable and, strain (UMD 2015 and susceptible), diet concentration, and the interaction as the fixed effects. If interaction term was significant, we performed post-hoc mean comparisons (α = 0.05; Bonferroni correction) of weight gain between UMD 2015 and susceptible strains for each level of the diet concentration.

### Fitness costs of resistance evolution

Previous attempts to establish a *H*. *zea* colony from surviving larvae collected from Cry1Ab sweet corn failed, possible due to suspected fitness costs (see S2 Appendix). To address the fitness issue, the UMD 2015 and susceptible strains were reared under laboratory conditions and assessed simultaneously for egg hatch, survival from egg to adult, development time from egg hatch to adult, and pupal weight. Methods and results of these studies are described in S2 Appendix.

### *Helicoverpa zea* population abundance and damage association

Year-to-year differences in *H*. *zea* population abundance over the study period could account for changes in control efficacy. To test this, we obtained archived blacklight trap data from the Maryland Department of Agriculture, which conducted a statewide monitoring network over the study period up to 2014 [55]. Additional trap data was obtained from the University of Delaware Insect Trapping Program [58] for years 2015 and 2016. Both data sets contained *H*. *zea* moth activity from traps at and near our farm locations. We calculated nightly mean moth numbers for each year by averaging across multiple trap sites, over a 14-day period starting at the onset of silking at each planting.

We analysed trends in *H*. *zea* population abundance at the sites, and its association with damages for Bt hybrids, through LMMs based on REML. For *H*. *zea* abundance, the LMM included mean nightly moth captures as response, year as fixed effect, and study site as random effect to account for repeated measurement. For LMM analysing the relationship between *H*. *zea* population abundance and damage in Bt hybrids, damaged corn ears (percentage) for Bt hybrids was treated as the response, mean nightly *H*. *zea* abundance as predictor, and site as random variable to account for repeated measures.

### Efficacy and tissue toxicity comparisons among the Bt hybrids

The same hybrids of Cry1A.105+Cry2Ab2 sweet corn were evaluated over each study period. However, different Cry1Ab hybrids were planted over the study period as they became commercially available and widely used by growers. Plots were planted with Bt hybrid ‘GSS0937’ and its isoline ‘Bonus’ during 1996–1997, Bt hybrid ‘GSS0966’ and its isoline ‘Prime Plus’ during 1998–2001, and Bt bicolor hybrid ‘BC0805’ and its isoline ‘Providence’ for the remainder years (2002–2014). To explore the possibility that differences in toxin expression among hybrids could account for changes in control efficacy, field performance of six Cry1Ab hybrids and the non-Bt expressing hybrid Providence were evaluated in 2008. Hybrids included BC0805 and GSS0966, along with newer hybrids (BSS0977, BSS0982, WH0809, and WH0812) that were not used in the study. The GSS0937 hybrid planted during the first two years was not included because seed was not available. Five replicate blocks of the seven hybrids (four at Queenstown, one at Upper Marlboro) were planted over a three-week period during June to provide silking stages attractive to a range of *H*. *zea* moth pressure during the late summer. To synchronize the silking period among hybrids, plantings in each block were staggered over several days according to the expected maturity time of each hybrid. Plots consisted of four rows spaced 90 cm apart and 15 m long, and were managed according to recommended commercial practices including overhead irrigation but no insecticide applications. Replicate blocks of hybrids were harvested at fresh market maturity on August 22, September 8, 18, and 24, and October 7. Data were recorded (as described above) on the percentage of ears damaged, mean instar stage, and extent of kernels consumed by each insect pest by examining 100 ears per plot.

We compared the rate of damage, instar and consumption among the six Bt hybrids and non-Bt hybrid ‘Providence’ using LMMs based on REML, assuming normal errors. We constructed separate LMMs, each with damage, instar and consumption as response variables, variety as the predictor, and site as a random effect to account for the site level differences. Tukey’s HSD tests (α = 0.05) was used to identify significant differences in the pairwise comparisons of the estimated means.

We further tested for potential differences in Cry1Ab toxicity (possibly due to expression) among the seven hybrids. Tissue samples of fresh green silk, wilted silk (after pollination), brown silk (at harvest), and kernel tissue (at harvest) were collected from the field plots at 2, 7, 18, and 18 days after the onset of silking, respectively. The frozen tissue material was lyophilized and processed into a fine powder in a tissue grinder, and stored at -80°C until further usage. The level of biological activity of Cry1Ab in each tissue type was determined by a diet-incorporated feeding bioassay with neonate *H*. *zea* as the sensitive indicator. We obtained susceptible *H*. *zea* eggs from a laboratory colony reared over many generations at the U.S. Department of Agriculture—Agricultural Research Service, Corn Insects and Crop Genetics Research Unit in Ames, Iowa. Eggs were incubated in an environmental chamber under temperature regimes manipulated to schedule a supply of test larvae for each replicate bioassay. The powdered tissue type from each hybrid was mixed in a *H*. *zea* meridic diet (Southland Products, Lake Village, AR) at a concentration of 3 g per liter of diet, which results in statistically detectable responses of body weight gain with little mortality [44].

Neonate *H*. *zea* were reared individually in Bio-Serv 128-cell clear plastic bioassay trays with clear film lid covers, with each cell containing 1.5 ml of diet. The larvae were allowed to feed for seven days, after which data were recorded on the weight and instar stage of surviving larva. The percentage of growth inhibition was calculated based on the difference in body weight relative to the weight of larvae fed on non-expressing tissue of each hybrid. In total, we tested 128 larvae in each of four replicate bioassays of each tissue type, collected from the corresponding replicate field blocks. *H*. *zea* body weight, growth inhibition, and instar stage among different Bt hybrids relative to the non-Bt expressing hybrid served as indicators of potential differences in the tissue toxicity.

Differences among Bt hybrids and the non-Bt hybrid for *H*. *zea* weight and growth inhibition were analysed by GLM assuming a Gaussian error distribution and identity link function. Tukey’s HSD tests (α = 0.05) identified significant differences in the pairwise comparisons of the GLM estimated means. We used diagnostic plots visualizing within-group residuals (standardized residuals Vs fitted values, normal Q-Q plots, histograms of residuals) and estimated random effects (normal Q-Q plots and pairs-scatter plot matrix) to ensure LMM appropriateness (see [59]; pgs. 174–197). Package ‘lme4’ [60] was used to construct the LMMs / GLMM, multiple comparison contrasts and Tukey’s HSD comparisons were performed with packages “contrast” and “multcomp” [61,62]. Estimated coefficients were extracted from the LMMs and plotted using “ggplot2” [63], all in R program [64].

## Results

### Control efficacy assessments

**Fig 1:**
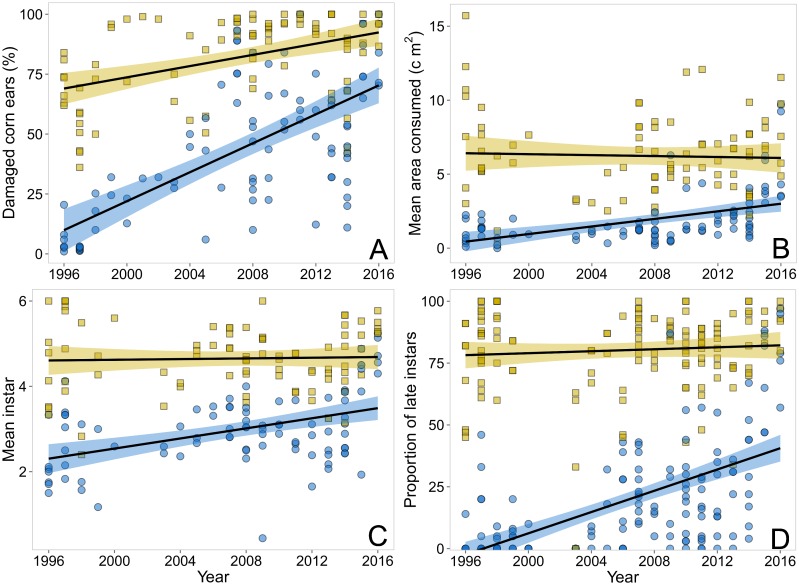
Trends comparing efficacy of Cry1Ab Bt sweet corn (Attribute) with non-Bt isogenic hybrids for control of *Helicoverpa zea* during 1996–2016 in Maryland, USA. Over years, Bt hybrids (blue circles and shades) had significant increases in the percentage of *H*. *zea* damaged ears (A), kernel consumption (B), instar stage (C) and proportion of late instars (D) than non-Bt hybrids (yellow squares and shades). Points represent the raw data, black lines represent the predictions from LMMs, while shaded region denote the upper and lower confidence levels (95% CI).

Data from Cry1Ab hybrids and the non-Bt counterparts were analysed for *H*. *zea* development and ear damage. During all years, *H*. *zea* infestations and ear damage were very high in the non-Bt hybrids, averaging 82.4±17.5% damaged ears, with 5.13±2.6 cm^2^ of kernel area consumed by larvae consisting of 80.3±17.6% late instars. Trends in these responses over years showed either no change or slight increases. Comparing trends between Bt and non-Bt hybrids, LMMs revealed significant interactions between year and hybrid type for all response variables denoting significant slope differences—damage [F _(1,175)_ = 18.7, *P* < 0.001]; consumption [F _(1,167)_ = 12.6, *P* <0.001]; instars [F _(1,169)_ = 9.4, *P* = 0.009] and proportion of late instars [F _(1,281)_ = 94.1, *P* < 0.001] (model coefficients are available in Table 1 of S3 Appendix). Changes over time in damage, consumption, instars and proportion of late instars were significantly different between Bt and non-Bt hybrids, with steeper increases for Bt than non-Bt hybrids (Fig 1A–1D). Over the 21 years, Cry1Ab hybrids experienced a greater change (88% relative increase) in ear damage by *H*. *zea* than non-Bt hybrids (Fig 1A), with 6.3% of the ears damaged in 1996 compared to 85.1% damaged ears in 2016 (based on raw averages). Since 1996, mean area of Bt kernels consumed per ear increased from 0.6 cm^2^ to about 3 cm^2^ (Fig 1B), while area of non-Bt hybrid consumption remained about the same or had declined marginally. The mean instar recorded in Bt hybrids also increased over the study period, indicating that surviving larvae reached later developmental stages (Fig 1C), while there was relatively no change for non-Bt hybrids. In particular, the proportion of late instars in Bt hybrids increased markedly over the past decade compared to that of non-Bt hybrids. Relatively few surviving larvae reached the 4^th^ instar in Bt ears during 1996–2006, while 37% of the surviving larvae were 4^th^-6^th^ instars during 2015–2016 (Fig 1D).

**Fig 2:**
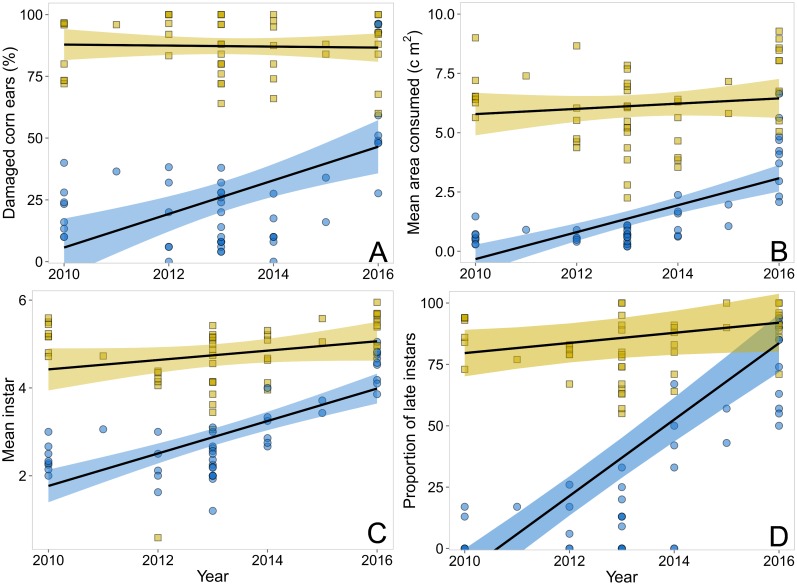
Trends comparing efficacy of Cry1A.105+Cry2Ab2 Bt sweet corn (Performance Series) with non-Bt isogenic hybrids for control of *Helicoverpa zea* during 2010–2016 in Maryland, USA. Over years, Bt hybrids (blue circles and shades) had significant increases in the percentage of *H*. *zea* damaged ears (A), kernel consumption (B), instar stage (C) and proportion of late instars (D) than non-Bt hybrids (yellow squares and shades). Points represent the raw data, black lines represent the predictions from LMMs / GLMM, while shaded region denote the upper and lower confidence levels (95% CI).

Similar trends are evident in Fig 2 showing increasing *H*. *zea* infestations and ear damage resulting in reduced field efficacy of the Cry1A.105+Cry2Ab2 sweet corn hybrids. Of the 50 plantings evaluated during 2010–2016, LMMs revealed significant interactions between year and hybrid type for all response variables denoting significant steeper slopes for the Bt hybrids—damage [F _(1,93)_ = 18.9, *P* < 0.001]; consumption [F _(1,90)_ = 14.4, *P* <0.001]; instars [F _(1,91)_ = 12.9, *P* <0.001] and proportion of late instars [X^2^ = 67.9, *P* < 0.001] (model coefficients are available in Table 2 of S3 Appendix). Ear damage (mean 87.1±11.2%), kernel consumption (mean 5.5±1.79 cm^2^) and the proportion of late instars (mean 83.4±12.2%) in the isogenic non-Bt hybrids showed only slight increases or no significant change over the seven years (Fig 2). Damaged ears of Cry1A.105+Cry2Ab2 sweet corn averaged 20.2±12.4% during 2010–2012, with 0.13±0.11 cm^2^ of kernel area consumed by larvae consisting of only 6.4±8.69% late instars. The field efficacy rapidly changed during 2015–2016 when damaged ears increased to 59.5 ±27.6% and kernel area consumed increased to 2.5±1.9 cm^2^, with 70.4±17.5% of the surviving *H*. *zea* reaching late instars (Fig 2A–2D).

Data from 13 plantings of Vip3A + Cry1Ab sweet corn evaluated during 2008–2016 were not analysed because no live larvae and no ear damage were found. The ears of non-Bt isogenic hybrids, paired with these Bt plots at each planting, experienced high *H*. *zea* infestations, resulting in 87.9±14.1% damaged ears and 5.4±2.4 cm^2^ kernel area consumed. Under this high pressure, the pyramided hybrids expressing Vip3A + Cry1Ab toxins provided 100% control of *H*. *zea*, as well as fall armyworm and European corn borer.

### Bioassays for resistance characterization

**Fig 3:**
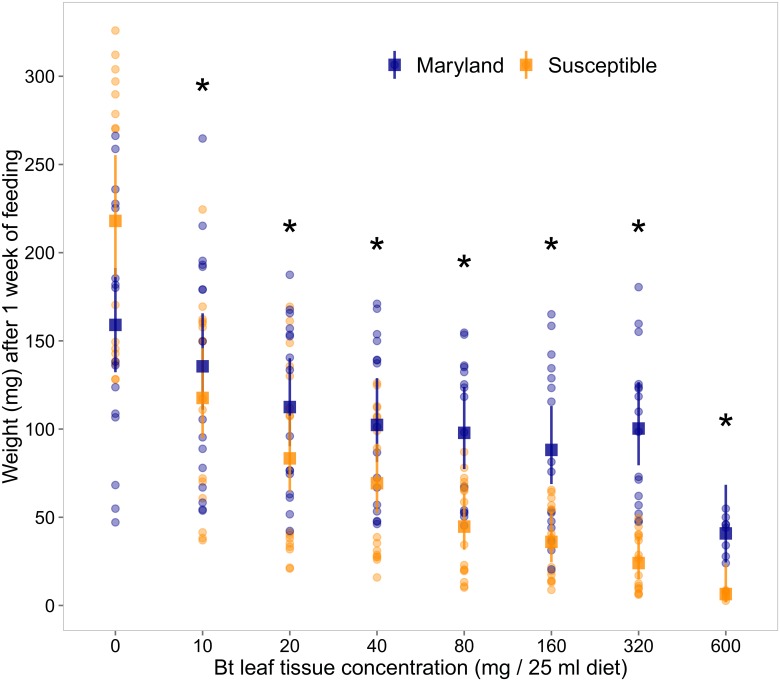
Comparison of weight between susceptible laboratory and field collected Maryland populations of *Helicoverpa zea* across concentrations of Cry1Ab expressing green leaf tissue. At every diet concentration other than control (dosage = 0), weight of larvae (mg) after 1 week of feeding assays was significantly higher for the Maryland strain (blue), than the susceptible (orange) strain. Points represent the raw data values; while the squares represent quasi-Poisson GLM estimated means, and the vertical lines denoting the confidence intervals around the mean. Asterisks indicate statistically significant differences (*P* < 0.05) based on post-hoc comparisons with a Bonferroni correction.

When fed diet without leaf powder, larval weight gain (218.0±19.92 mg) of the susceptible strain was 27% more than that of the UMD 2015 strain (159.1±17.9 mg). Although not significantly different [*F*_(1,3)_ = 5.57, *P* = 0.099], this suggests that the susceptible strain may be more adapted to the artificial diet. However, leaf tissue-incorporated bioassay results showed a significant interaction between strain and tissue concentration for weight gain [X^2^ = 46.27, df = 1, *P* < 0.001]. Weight gain of the susceptible strain was significantly reduced at a greater rate than the UMD 2015 strain at all concentrations tested (Fig 3).

### *Helicoverpa zea* population abundance and damage association

**Fig 4:**
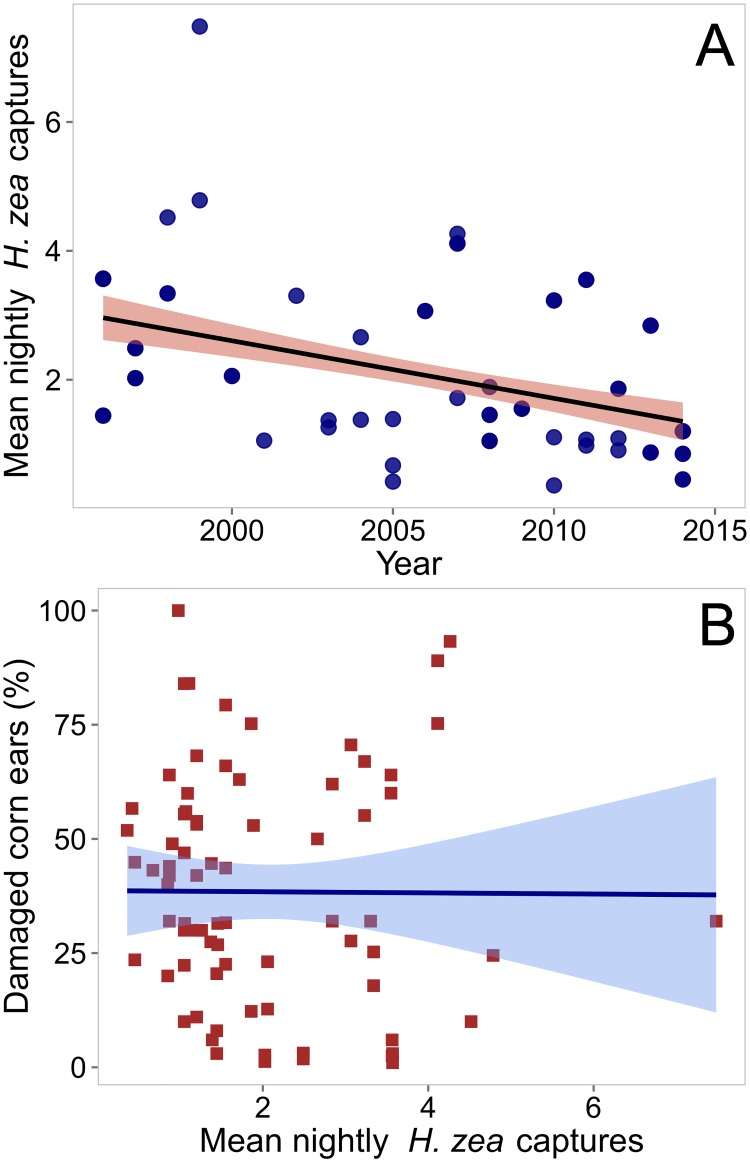
Mean nightly captures of *Helicoverpa zea* in black light traps during 1996–2014 in Maryland, USA and its association with damage to Bt corn hybrids. The population abundance of *H*. *zea* at or near study sites decreased over the past two decades (A), and showed no relationship with damage levels in Bt sweet corn hybrids (B). Lines represent predictions from LMMs and shades depict upper and lower confidence intervals (95% CI).

The population abundance of *H*. *zea* during the 14-day period following silking at each planting decreased significantly over the study period from 3 moths captured per night in 1996 to 1.3 in 2014 [*F*_(1, 165)_ = 48.7, *P*<0.001; slope = -0.10; Fig 4A]. Additional data from 12 trap locations on the Delmarva Peninsula showed even lower *H*. *zea* moth activity, with captures per night averaging 0.64±0.48 in 2015 and 0.42±0.45 in 2016. *H*. *zea* population abundance did not significantly affect the observed damages to corn ears for Bt hybrids [*F*_(1, 82)_ = 0.001, *P* = 0.97; Fig 4B].

### Efficacy and tissue toxicity comparisons among the Bt hybrids

**Fig 5:**
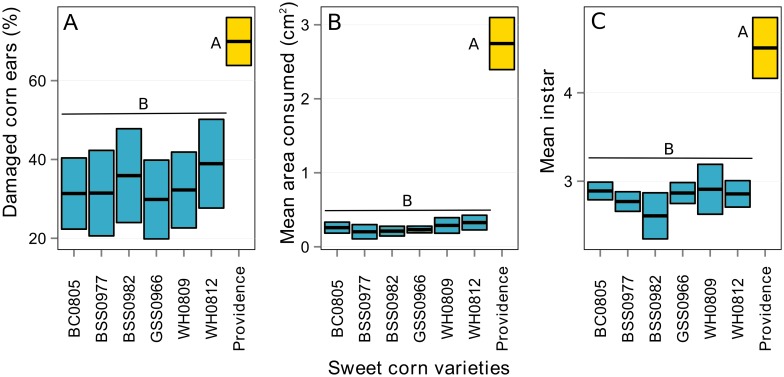
Results comparing control efficacy variables among Bt and non-Bt hybrid varieties in Maryland, USA in 2008. The mean damaged ears (A), kernel area consumed, (B), and instars of susceptible larvae (C) was broadly similar among the Cry1Ab expressing Bt hybrids (blue bars), and significantly different (Tukey’s HSD; α = 0.05; denoted by unique alphabets) from non-Bt control hybrids (yellow bar). Black lines represent the mean values and the height of bars above and below the black lines respectively indicate the upper and lower confidence levels (95% CI) estimated through LMMs.

**Fig 6:**
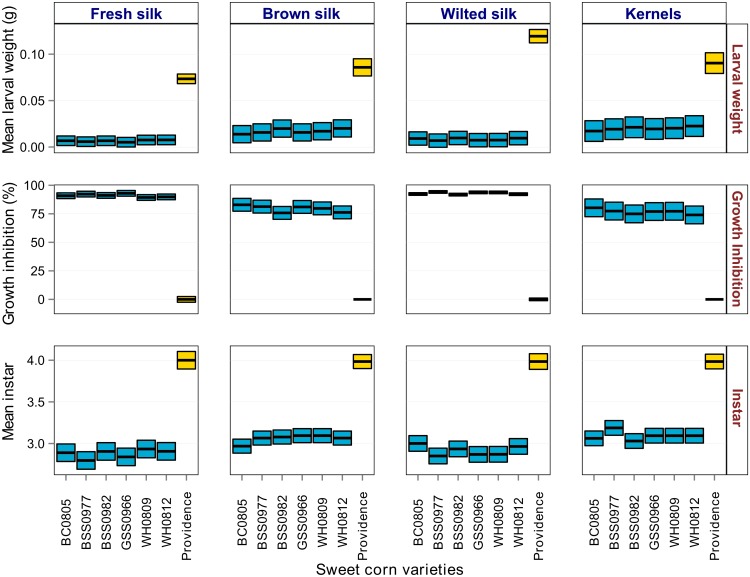
Results comparing control efficacy in relation to tissue toxicity among Bt and non-Bt sweet corn varieties from Maryland, USA in 2008. The mean weight, growth inhibition and instars of susceptible larvae feeding on different sweet corn tissues was broadly similar among the Cry1Ab expressing Bt hybrids (blue bars), and significantly different (Tukey’s HSD; α = 0.05) from non-Bt control hybrids (yellow bar). Black lines represent the mean values and height of bars indicate the upper and lower confidence levels (95%CI) estimated through Gaussian GLMs or ANOVA.

The seven hybrids within each replicate block reached the silking stage within several days of each other and thus were exposed similarly to the oviposition pressure by *H*. *zea*. High ear infestations in the non-Bt hybrid caused an overall 70% damaged ears and 2.75 cm^2^ of kernel consumption, and larvae found in ears averaged 4.5 instar stage in developmental growth. Values of damage, consumption, and mean instars across the Bt hybrids were significantly less than the non-Bt control hybrid but did not vary significantly among the Bt hybrids (Fig 5A–5C; see Table 3 in S3 Appendix for statistical tables). The mean weight of larvae feeding on different corn tissues was similar among the Bt hybrids and significantly less than non-Bt control hybrids (Fig 6). Inhibition of larval growth upon feeding on different tissues was also not significantly different among Bt hybrids but significantly higher than the non-Bt hybrid (Fig 6). The mean instar feeding on different sweet corn tissues was also similar among the Bt hybrids, and significantly less than the non-Bt hybrid (Fig 6). Brown dried silk and kernel tissue allowed 46% more weight gain and 4.5% more stadia development than that of green and wilted silk tissue, but there were no differences among the Bt hybrids.

## Discussion

Our field results clearly demonstrate the significantly increased susceptibility and reduced control efficacy of Cry1Ab Bt sweet corn (event Bt11) to *H*. *zea*, since its commercial introduction in 1996. We also report significant reductions in field performance of Cry1A.105+Cry2Ab2 sweet corn (event MON89034) for controlling *H*. *zea*, particularly during 2015–2016. Larvae successfully infested and damaged an increasingly large proportion of ears, consumed more kernel area, and reached later developmental stages (4^th^ - 6^th^ instars) in both events of Bt hybrids with increasing years of adoption. The control efficacy failure was unrelated to the *H*. *zea* population abundance. Moth activity monitored in blacklight traps declined over the past 21 years at the study sites. Among the different Cry1Ab hybrids, there was no evidence of tissue toxicity differences that could contribute to the decline in control efficacy. Field efficacy comparisons under high *H*. *zea* population pressure also showed that ear damage, consumption, and mean instars did not differ significantly among Cry1Ab expressing hybrids. Similarly, bioassay determinations of various plant tissues did not show significant Bt hybrid differences in *H*. *zea* body weight gains, percentage growth inhibition, or developmental larval stage. After ruling out these possible contributing factors, the rapid change in field efficacy in recent years and decreased susceptibility of *H*. *zea* to Bt sweet corn provide strong evidence of field-evolved resistance in *H*. *zea* populations to multiple Cry toxins. However, our field studies on Vip3A + Cry1Ab sweet corn (Attribute II) show no detectable change in *H*. *zea* susceptibility and confirm the high efficacy of this pyramided product reported earlier [50]. Our field results for Vip3A hybrids suggest that there may be no cross resistance with Cry1Ab, similar to previous reports of no cross-resistance for Vip3A with laboratory colonies of Cry1Ac resistant *H*. *zea* [38,39].

Results of our laboratory bioassays further demonstrate significant differences in weight gain and fitness characteristics between the susceptible strain and the UMD 2015 strain of *H*. *zea* reared from surviving late instars collected from Cry1Ab sweet corn. Although apparently more adapted to the artificial diet, weight gain of the susceptible strain significantly reduced at a greater rate than the UMD 2015 strain at all concentrations tested. Using Cry1Ab-expressing corn leaf powder Anilkumar *et al*. [38] showed significant, but marginal differences in weight loss (no differences in mortality) between susceptible and Cry1Ac-resistant *H*. *zea*. Fitness of the UMD 2015 strain was significantly lower than that of the susceptible strain as indicated by lower hatch rate, longer time to adult eclosion, lower pupal weight, and reduced survival to adulthood (see S2 Appendix). These results suggest that reduced fitness in the UMD 2015 strain is likely associated with changes in susceptibility to Cry toxins, which has been reported in other insects associated with resistance to Bt [27,65].

The fitness costs may also have broadly affected the timeframe of resistance evolution as well as the stability of resistance in the field. Previous reports identify sub-lethal effects of MON810 corn resulting in prolonged larval and pupal development, delayed adult eclosion and emergence, thereby resulting in asynchrony of mating between *H*. *zea* individuals [49,66]. Reports indicate that longer larval developmental period in resistant *S*. *frugiperda* has resulted in emergence asynchrony between adults susceptible and resistant to Cry1F (Bt maize event TC1507) in Puerto Rico [67]. Similarly, the prolonged larval and pupal development and later emergence of resistant adults (than susceptible strains) and resultant asynchrony in emergence and mating between resistant and susceptible strains could have delayed field-evolved resistance to Cry proteins in *H*. *zea*. Additionally, other fitness costs may have made it difficult for the resistant Maryland population to increase significantly to cause widespread field control failure earlier. Our previous unsuccessful three attempts at characterizing resistance evolution (see S1 Appendix) may be attributed to fitness costs associated with resistance development.

The field-evolved resistance we report here indicate ‘practical resistance’ (>50% resistant individuals and reduced efficacy [16,18]) given the probable genetic basis of resistance. Many sweet corn farmers in Maryland either have stopped growing Cry1Ab hybrids or are applying more insecticide sprays to compensate for the reduced control efficacy (personal communication, GPD). In 2016, there also have been numerous inquiries to seed companies from sweet corn farmers reporting control failures from Cry1A.105+Cry2Ab2 hybrids (personal communication, GPD). Our results confirm the findings from recent studies by Reisig and Reay-Jones [68,69] showing increases in kernel feeding by *H*. *zea* on Cry1A.105+Cry2Ab2 field corn (VT Double PRO) and evidence of developing resistance to the Cry1Ab trait based on changes over time in toxin inhibition on growth and development of *H*. *zea*.

Many factors could have contributed to development of *H*. *zea* resistance. The Cry1Ab toxin has been exerting selection pressure on *H*. *zea* populations in Bt corn since the trait became commercially available in 1996; however, we presume that the relatively low deployment of Bt sweet corn hybrids, planted without a refuge requirement, has unlikely exerted enough selection pressure alone to account for changes in *H*. *zea* susceptibility. Instead, the high adoption rate of Bt field corn and cotton, along with the moderate dose expression of Cry1Ab and related Cry1A toxins in these crops and decreasing refuge compliance, probably contributed significantly for evolution of resistance. Many field corn hybrids expressing Cry1Ab (events MON810 and BT11) or Cry1A.105+Cry2Ab2 (event MON89034) are widely used and the moderate dose expression could allow heterozygous resistant individuals containing minor resistance alleles to survive, thus increasing the frequency of genes that confer resistance in the *H*. *zea* population [13,70]. Regional deployment levels of Bt corn hybrids can also contribute to increasing resistant gene frequencies, with low technology adoption related to slower rate of resistance evolution [71]. The adoption rate of Bt field corn in Maryland is very high, accounting for 83–93% of the hectares planted during 2013 in crop reporting districts where the Queenstown and Salisbury sites are located. This high adoption of Bt corn, along with a presumed decline in compliance with the refuge requirements, has almost certainly contributed at the local level to the evolution of resistance. Similarly, Bt corn adoption exceeds 80% throughout most corn production areas in the U.S., and surveys show that 22% of the farmers are not complying with refuge requirements [72].

The behavior and life history characteristics of *H*. *zea* may have aided in evolution of resistance, similar to documented cases of evolution of resistance to conventional insecticides [73–75]. At eastern Maryland sites, *H*. *zea* overwinters successfully during most years and thus resistant individuals are able to contribute resistant alleles to the population in the subsequent year. In addition to local recruitment of resistant *H*. *zea* individuals, southerly flow weather patterns can facilitate the northward dispersal of moths, particularly as the maturing crops in the southern regions become less attractive as hosts [76]. This south-north migration of moths already selected for resistance on Bt corn and cotton can enhance the development of Cry1Ab resistance in the northern corn growing regions. Reisig and Reay-Jones [68] recently presented evidence of developing resistance to the Cry1Ab trait in field corn in the South and North Carolinas.

Behavioral changes in *H*. *zea* due to feeding on Bt corn can also increase the risk of evolution of resistance. Studies in Maryland reported that sublethal exposure to Cry1Ab toxin in Bt corn delayed larval development and adult emergence, which may result in asynchrony of mating between individuals emerging from Bt and non-Bt corn [66]. Another study showed that alterations in the cannibalistic behavior of *H*. *zea* larvae due to sublethal exposure to the Cry1Ab toxin could increase the selective differential between susceptible individuals and those carrying resistance genes [77]. The extent that each behavioral factor contributes to the current field efficacy failures and *H*. *zea* resistance to Cry1Ab is unknown, but collectively they probably have aided in the evolution of resistance.

The field-evolved resistance by *H*. *zea* has implications for resistance monitoring, IRM and the sustainability of the Bt corn technology. Registrants of Bt field corn expressing Cry1Ab, and Cry1A + Cry2Ab2 toxins are required by EPA to annually monitor populations of *H*. *zea* using laboratory bioassays to detect field-evolved resistance early enough to enable proactive management before field failures occur [43]. Maryland has historically represented the northern range of overwintering *H*. *zea* [54]. With these populations evolving resistance, programs monitoring for resistance in further northern ranges of *H*. *zea* are essential. Warming temperatures exacerbate the risk of resistance spreading, as latitudes north of 40° become conducive for successful overwintering and increasing migration of *H*. *zea* [15,78].

We predict that *H*. *zea* resistance to the Cry toxins is likely to increase, and spread, with the shift to RIB corn hybrids that contain a blend (e.g. 95:5 Bt: non-Bt seed). Similarly, due to northward influxes of potentially resistant moths from southern source regions, the risk of further evolution of resistance may increase with the reduced refuge size (from 50% to 20%) in regions where Bt cotton is used. Apart from reductions in the refuge size, there are also concerns that pollen-mediated gene flow between Bt and refuge plants in a seed blend could accelerate resistance evolution. Cross-pollination between refuge and neighboring Bt plants can result in fewer susceptible *H*. *zea* moths produced in a refuge or intermediate levels of toxin expressed in kernels that kill susceptible individuals but allow heterozygotes to survive [79,80]. The potential existence of cross-resistance between Cry1Ab and the other Cry toxins [13,35,39,81–83] may also compromise the efficacy and durability of the new pyramided traits in Bt field and sweet corn. Finally, as reported here and by Burkness *et al*. [50], Vip3A expression in Attribute II sweet corn and many field corn products is still highly effective for controlling *H*. *zea* and other lepidopteran pests. Although there is no reported cross-resistance between Vip3A and Cry toxins [38,81,84], it is unknown whether continued selection pressure from Cry expressing Bt corn and increased use of the Viptera trait will reduce the sustainability of this pyramided Bt technology.

## Supporting Information

S1 Appendix Experiments to characterize Bt resistance in *Helicoverpa zea* field-collected populations during 2008 to 2012. (DOCX)

S2 Appendix 2015 Bioassays to characterize fitness of Maryland field-collected populations of *Helicoverpa zea*. (DOCX)

S3 Appendix Tables presenting the summary of statistical results for all analyses. (DOCX)
