# Correction: Field-Evolved Resistance in Corn Earworm to Cry Proteins Expressed by Transgenic Sweet Corn

**DOI:** 10.1371/journal.pone.0183637

**Published:** 2017-08-17

**Authors:** Galen P. Dively, P. Dilip Venugopal, Chad Finkenbinder

There is an error in Table 2 of the file S3 Appendix in the Supporting Information files. The terms “None” and “Square root” are switched under the headings in the first column, rows 3 and 4. Please see the complete, correct Table 2 here.

**Table 2:** Summary of model, data transformations, fixed effects, parameters and coefficients of response variables comparing trends in ear damage and infestations of *Helicoverpa zea* in Cry1A.105+Cry2Ab2 Bt and nonBt sweet corn hybrids during 2010–2016 in Maryland. The negative coefficient estimates for the interaction parameter (Year: TreatmentNon-Bt) indicates that the rate of changes (damage, consumption, instar growth and late instar proportion) over the past 7 years was lesser for non-Bt than Bt varieties.

Response variable/ model/ data transformation	Fixed effects	Model parameters	Coefficient estimate	Standard error
Damaged ears (%) LMM Square root	Year + Treatment (Bt vs non-Bt) + Year: Treatment	Intercept	2.41	0.52
		Year	0.72	0.12
		TreatmentNon-Bt	7.08	0.63
		Year: TreatmentNon-Bt	-0.74	0.17
Mean area consumed (cm^2^) LMM None	Year + Treatment (Bt vs non-Bt) + Year: Treatment	Intercept	-0.87	0.56
		Year	0.77	0.10
		TreatmentNon-Bt	6.46	0.52
		Year: TreatmentNon-Bt	-0.53	0.14
Mean instar LMM Square root	Year + Treatment (Bt vs non-Bt) + Year: Treatment	Intercept	1.23	0.08
		Year	0.14	0.02
		TreatmentNon-Bt	0.77	0.09
		Year: TreatmentNon-Bt	-0.09	0.02
Proportion of late instars Gaussian GLMM (identity link function) None	Year + Treatment (Bt vs non-Bt) + Year: Treatment	Intercept	-25.25	5.55
		Year	15.56	1.18
		TreatmentNon-Bt	102.79	6.15
		Year: TreatmentNon-Bt	-13.49	1.64
