# Supplementary material for: Field-Evolved Resistance in Corn Earworm to Cry Proteins Expressed by Transgenic Sweet Corn
Source: PLoS One. 2016 Dec 30;11(12):e0169115. doi: 10.1371/journal.pone.0169115 (PMC5201267; doi:10.1371/journal.pone.0169115)
Supplement: S1 Appendix — (DOCX) [file pone.0169115.s001.docx]

**S1 Appendix. Experiments to characterize Bt resistance in *Helicoverpa zea* field-collected populations during 2008 to 2012.**

On three separate occasions, surviving fifth and sixth instar *H. zea* were collected from ears in Cry1Ab-expressing sweet corn plots during late August from the Salisbury study site on the Eastern Shore and reared individually on meridic diet until pupation. These collections were shipped to several laboratories, where colonies of moths were maintained to produce eggs, and attempts to characterize Bt resistance in *H. zea* used bioassay procedures similar to those used to determine baseline susceptibility of *H. zea* and *O. nubilalis* to the Cry1Ab toxin [1].

In 2008, 180 pupae reared from larvae were shipped to Dr. Bill Moar’s Laboratory at Auburn University for resistance screening. Of these pupae, about one third were infected with various pathogens (primarily nuclear polyhedrosis virus and fungal pathogens) and died. Moth emergence was very low, resulting in 10 pairings of male and female moths that produced only infertile eggs. To increase the fitness of the breeding colony, male moths were mated with females from a susceptible laboratory population to produce enough fertile eggs in order to increase the colony size. Beginning at the F2 generation, larvae were selected for Cry1Ab resistance by gradually increasing the exposure dose in a feeding bioassay with toxin-incorporated diet. A 70-fold resistance ratio was observed in the induced colony after 8 generations of selection, but the colony collapsed in June 2009.

In 2010, late instar *H. zea* were again collected at the Salisbury site and produced about 800 pupae, of which 200 were shipped to Bruce Lang (Custom Bio-Products, Maxwell, IA), a third party laboratory contracted by the seed companies to monitor shifts in *H. zea* susceptibility to the Cry1Ab toxin, and 600 were shipped to the Syngenta laboratory (Research Triangle Park, NC) for testing. Similar rearing problems were reported by these collaborators, where seemingly healthy adult moths emerged from less than 20% of the pupae but colonies failed to lay enough fertile eggs to conduct a full series bioassay. Finally, about 1,500 late instars were collected at the Salisbury site in 2011 and 2012, and reared to moth emergence at the University of Maryland. Attempts to establish a Bt resistant laboratory colony also failed because of low fecundity and infertile eggs, leading to colony collapse or loss of resistance characteristics after 3 generations.

References

1. Siegfried BD, Spencer T, Nearman J (2000) Baseline susceptibility of the corn earworm (Lepidoptera: Noctuidae) to the Cry1Ab toxin from *Bacillus thuringiensis*. *J Econ Entomol* 93(4):1265–1268.
