# Supplementary material for: Field-Evolved Resistance in Corn Earworm to Cry Proteins Expressed by Transgenic Sweet Corn
Source: PLoS One. 2016 Dec 30;11(12):e0169115. doi: 10.1371/journal.pone.0169115 (PMC5201267; doi:10.1371/journal.pone.0169115)
Supplement: S2 Appendix — (DOCX) [file pone.0169115.s002.docx]

**S2 Appendix. 2015 Bioassays to characterize fitness of MD field-collected populations of *Helicoverpa zea*.**

Fitness bioassays were conducted at the University of Maryland using third generation eggs of the UMD 2015 strain and the susceptible laboratory strain of *H. zea* provided by Benzon Research (see S2 Appendix for rearing methods). Eighteen cohorts of eggs (less than 24 hours old) of each strain were reared in 73.9 ml plastic soufflé pots supplied with 10 ml of *H. zea* meridic diet. The pots were held in a growth chamber at 25°C, L:D 14:10, and 40-60% RH. Each cohort consisted of 20 to 40 eggs attached to a small section cut from the muslin fabric that was used as an oviposition substrate in colony rearing. After hatching was complete, the number of neonates was recorded and the larvae were then allowed to develop to the early 3rd instar. At that time, surviving larvae were transferred to 30 ml Solo plastic cup with ventilated lids and reared on meridic diet until adult eclosion. Daily observations tracked the development of each cohort and recorded data to measure percent egg hatch, survival from egg to adult, development time from egg hatch to adult, and pupal weight. One-way ANOVA was used to test for significant differences between means of each fitness parameters.

**Results.** Bioassays characterizing the fitness costs revealed significant differences between the susceptible and UMD 2015 strains for all fitness parameters measured. The UMD 2015 strain from *H. zea* larvae surviving on Cry1Ab-expressing sweet corn had significantly fewer egg hatchings [F _(1, 49)_ = 30.0, *P* <0.001], adult eclosion took longer [F _(1, 7)_ = 45.6, *P* <0.001], fewer survived to adulthood [F _(1, 7)_ = 74.6, *P* <0.001] and the pupae weighed less [F _(1, 81)_ = 7.65, *P* = 0.007] than the susceptible laboratory strain (Figs. 1A-D, respectively). The results show that there were significant fitness costs associated with the change in Bt susceptibility indicting resistance development (see S2 Appendix for toxicity responses).


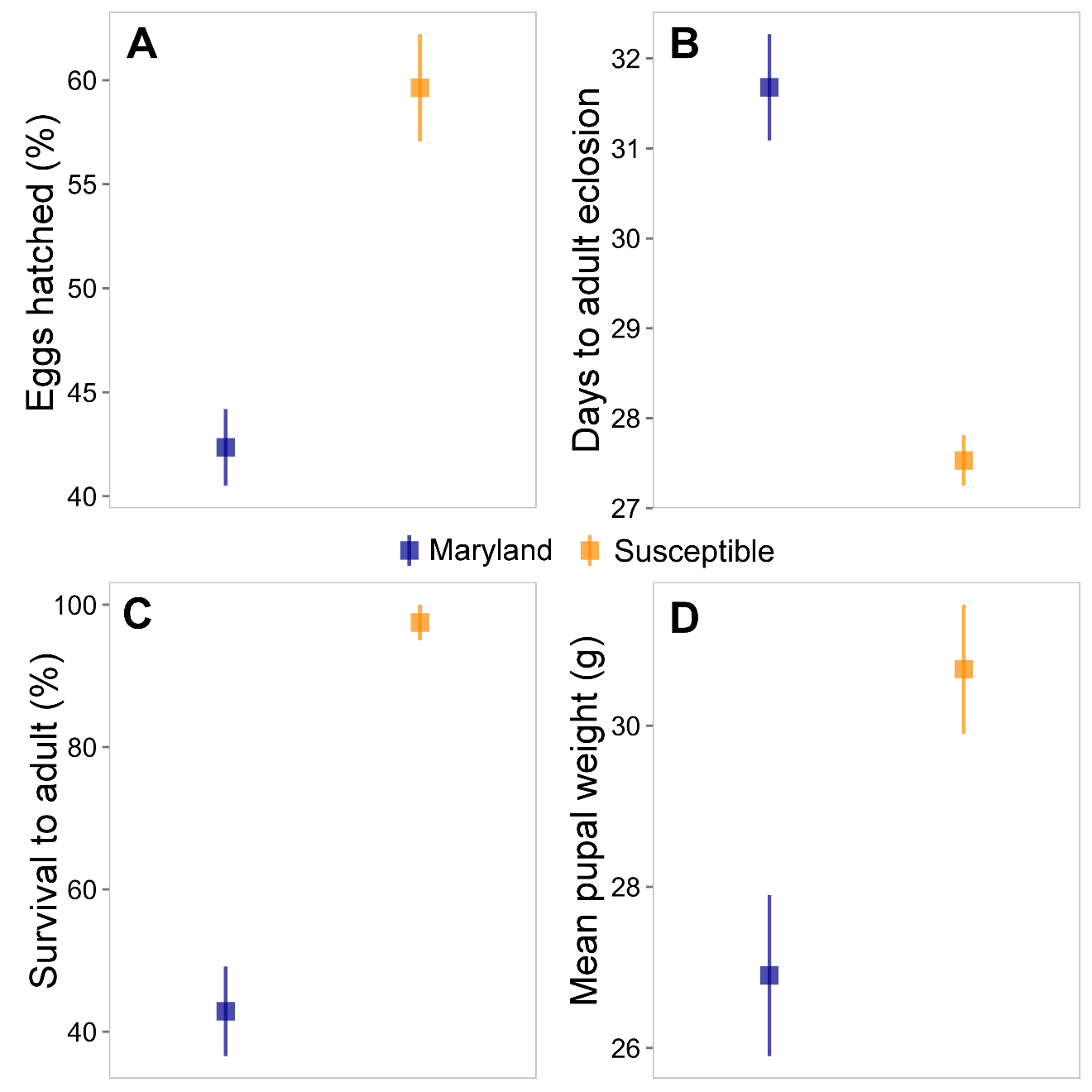


**Figure 1.** **Comparison of fitness parameters of a Maryland colony of *Helicoverpa zea* reared from larvae surviving Cry1Ab-expressing sweet corn compared to a susceptible laboratory strain.** ANOVA results showed that each of the measured fitness parameters – percentage eggs hatched (A), days to adult eclosion (B), percentage survival to adult (C) and mean pupal weight (D) – were significantly different (*P* < 0.01) between the MD (blue) and susceptible (orange) strains. Plots show the ANOVA estimated mean values (squares) and the associated standard errors (vertical lines).
