# Supplementary material for: Field-Evolved Resistance in Corn Earworm to Cry Proteins Expressed by Transgenic Sweet Corn
Source: PLoS One. 2016 Dec 30;11(12):e0169115. doi: 10.1371/journal.pone.0169115 (PMC5201267; doi:10.1371/journal.pone.0169115)
Supplement: S3 Appendix — (DOCX) [file pone.0169115.s003.docx]

**S3 Appendix. Tables presenting the summary of statistical results for all analyses.**

**Table 1. Summary of model, data transformations, fixed effects, parameters and coefficients of response variables comparing trends in ear damage and infestations of *Helicoverpa zea* in Cry1Ab Bt and nonBt sweet corn hybrids during 1996-2016 in Maryland.** The negative coefficient estimates for the interaction parameter (Year: TreatmentNon-Bt) indicates that the rate of changes (damage, consumption, instar growth and late instar proportion) over the past 21 years was lesser for non-Bt than Bt hybrids.

| **Response variable/**  **model/ data transformation** | **Fixed effects** | **Model parameters** | **Coefficient**  **estimate** | **Standard error** |
| --- | --- | --- | --- | --- |
| Damaged ears (%)  LMM  None | Year +  Treatment (Bt vs non-Bt) + Year : Treatment | Intercept | 7.17 | 4.38 |
|  |  | Year | 2.96 | 0.30 |
|  |  | TreatmentNon-Bt | 61.08 | 5.82 |
|  |  | Year : TreatmentNon-Bt | -1.82 | 0.42 |
| Mean area consumed (cm^2^)  LMM  Square root | Year +  Treatment (Bt vs non-Bt) + Year : Treatment | Intercept | 0.60 | 0.15 |
|  |  | Year | 0.04 | 0.01 |
|  |  | TreatmentNon-Bt | 1.69 | 0.16 |
|  |  | Year : TreatmentNon-Bt | -0.04 | 0.01 |
| Mean instar  LMM  None | Year +  Treatment (Bt vs non-Bt) + Year : Treatment | Intercept | 2.34 | 0.19 |
|  |  | Year | 0.04 | 0.01 |
|  |  | TreatmentNon-Bt | 2.31 | 0.24 |
|  |  | Year : TreatmentNon-Bt | -0.05 | 0.02 |
| Proportion  of late instars  LMM  Square root | Year +  Treatment (Bt vs non-Bt) + Year : Treatment | Intercept | -1.06008 | 0.62 |
|  |  | Year | 0.34 | 0.02 |
|  |  | TreatmentNon-Bt | 8.69 | 0.23 |
|  |  | Year : TreatmentNon-Bt | -0.28 | 0.03 |

**Table 2. Summary of model, data transformations, fixed effects, parameters and coefficients of response variables comparing trends in ear damage and infestations of *Helicoverpa zea* in Cry1A.105+Cry2Ab2 Bt and nonBt sweet corn hybrids during 2010-2016 in Maryland.** The negative coefficient estimates for the interaction parameter (Year: TreatmentNon-Bt) indicates that the rate of changes (damage, consumption, instar growth and late instar proportion) over the past 7 years was lesser for non-Bt than Bt varieties.

| **Response variable/**  **model/ data transformation** | **Fixed effects** | **Model parameters** | **Coefficient**  **estimate** | **Standard error** |
| --- | --- | --- | --- | --- |
| Damaged ears (%)  LMM  None | Year + Treatment (Bt vs non-Bt) + Year : Treatment | Intercept | 2.41 | 0.52 |
|  |  | Year | 0.72 | 0.12 |
|  |  | TreatmentNon-Bt | 7.08 | 0.63 |
|  |  | Year : TreatmentNon-Bt | -0.74 | 0.17 |
| Mean area consumed (cm^2^)  LMM  Square root | Year + Treatment (Bt vs non-Bt) + Year : Treatment | Intercept | -0.87 | 0.56 |
|  |  | Year | 0.77 | 0.10 |
|  |  | TreatmentNon-Bt | 6.46 | 0.52 |
|  |  | Year : TreatmentNon-Bt | -0.53 | 0.14 |
| Mean instar  LMM  None | Year + Treatment (Bt vs non-Bt) + Year : Treatment | Intercept | 1.23 | 0.08 |
|  |  | Year | 0.14 | 0.02 |
|  |  | TreatmentNon-Bt | 0.77 | 0.09 |
|  |  | Year : TreatmentNon-Bt | -0.09 | 0.02 |
| Proportion  of late instars  Gaussian GLMM  (identity link function)  None | Year + Treatment (Bt vs non-Bt) + Year : Treatment | Intercept | -25.25 | 5.55 |
|  |  | Year | 15.56 | 1.18 |
|  |  | TreatmentNon-Bt | 102.79 | 6.15 |
|  |  | Year : TreatmentNon-Bt | -13.49 | 1.64 |

**Table S3. Summary of statistical results comparing efficacy and tissue toxicity among the Bt and non-Bt sweet corn hybrids during 2008.** The efficacy comparisons are based on ANOVA, while Gaussian GLM was used for the tissue toxicity comparisons. Seven sweet corn varieties were compared in total (6 Bt and 1 non-Bt), hence for all these models the degrees of freedom and residual degrees of freedom were 6 and 21 respectively.

| Analysis | Response variable | Tissue | F-value | p-value |
| --- | --- | --- | --- | --- |
| Efficacy comparisons | Damaged ears (%) | NA | 4.59 | 0.0022 |
|  | Area consumed (cm) | NA | 50.13 | <.0001 |
|  | Instars | NA | 8.95 | <.0001 |
| Tissue toxicity comparisons | Larval weight (g) | Fresh silk | 91.33 | <.0001 |
|  |  | Brown silk | 30.89 | <.0001 |
|  |  | Wilted silk | 127.42 | <.0001 |
|  |  | Kernels | 21.88 | <.0001 |
|  | Growth inhibition (%) | Fresh silk | 762.83 | <.0001 |
|  |  | Brown silk | 114.05 | <.0001 |
|  |  | Wilted silk | 4321.8 | <.0001 |
|  |  | Kernels | 68.04 | <.0001 |
|  | Instars | Fresh silk | 79.46 | <.0001 |
|  |  | Brown silk | 66.38 | <.0001 |
|  |  | Wilted silk | 93.551 | <.0001 |
|  |  | Kernels | 57.90 | <.0001 |
